# Understanding How Immigrant Fertility Differentials Vary over the Reproductive Life Course

**DOI:** 10.1007/s10680-019-09536-x

**Published:** 2019-09-30

**Authors:** Ben Wilson

**Affiliations:** 1 Department of Sociology, Stockholm University, 106 91 Stockholm, Sweden; 2 Department of Methodology, London School of Economics, Houghton St, London, WC2A 2AE UK

**Keywords:** Immigrant, Fertility, Childbearing, Life course, Differentials, UK

## Abstract

Studies of immigrant fertility differentials indicate that foreign-born women have more children than native-born women, at least for some origin groups. Yet little is known about variation in cumulative fertility differentials over the life course, including the extent to which this variation develops into completed fertility differentials. This research responds with an analysis of cumulative fertility differentials in the UK for a cohort of women born between 1942 and 1971. Findings are consistent with age-specific patterns that have been documented for immigrant groups in the UK, but underline the importance of taking a cohort perspective, which helps to distinguish between the tempo and quantum of fertility. Immigrants have significantly higher completed fertility than UK-born natives if they were born in India, Pakistan, Bangladesh, Jamaica, or Western and Central Africa, but the profile of their cumulative fertility differentials—versus the UK-born—varies considerably over the life course, especially by age at migration. For example, women from Bangladesh and Pakistan have similar levels of cumulative fertility at age 40, but very different age patterns of cumulative fertility from ages 20–40. There is a consistent pattern of relatively delayed Pakistani fertility at early ages, especially for those arriving at later ages, but the same is not true for women from Bangladesh. Overall, these results imply that researchers should beware of variation in cohort fertility over the life course—with respect to both the quantum and tempo of fertility—when analysing immigrant childbearing, in addition to variation by origin and age at arrival.

## Introduction

One of the most common aims of research on migrant fertility is to understand the differences between foreign-born and native-born fertility; often referred to as *immigrant fertility differentials*. Research suggests that these differentials exist in almost all high income countries, especially those in Europe, North America, and Oceania (e.g. Abbasi-Shavazi and McDonald 2000; Adserà and Ferrer 2014b; Bélanger and Gilbert 2006; Haug et al. 2002; Parrado and Flippen 2012; Sevak and Schmidt 2008; Sobotka 2008; Statistics New Zealand 2012). Immigrant fertility differentials are of interest to demographers for a variety of reasons, not least because they help to understand the contribution of immigrants to population change in a destination country. This contribution is typically of interest in high income countries due to concerns about population ageing, which relate to pensions, old-age support ratios, and the proportion of the population that is of working age (Grant et al. 2004; Harper and Hamblin 2014). Not only do immigrants contribute to a destination’s population size via their number of children (and their absolute numbers, which are both related to the historical period when the immigration took place), but they also have an impact on population composition, especially the future age distribution of a population, via the timing of their births.

In addition to broader interests in population dynamics, researchers often analyse immigrant fertility differentials with a focus on either fertility or migration. This includes studies of the determinants of fertility, where immigrants are often compared to natives in an effort to understand how exposure to cultural and socio-economic norms influences childbearing behaviour (e.g. Bean and Swicegood 1985; Haug et al. 2002; Hill and Johnson 2004). Similarly, research often compares immigrant and native fertility to test a variety of hypotheses about migration and migrant fertility (Milewski 2010). This includes hypotheses that make predictions about the links between fertility and the timing of migration—for example *disruption* or the *interrelation of events* (e.g. Milewski 2007; Stephen and Bean 1992). It also includes hypotheses like *adaptation* and *intergenerational assimilation* that make predictions about fertility convergence, where convergence describes the way that differentials are expected to change over time (e.g. Kahn 1988; Parrado and Morgan 2008).

Despite the importance of immigrant fertility differentials for each of these research interests, there is a lack of research that shows how these differentials accumulate over the reproductive life course (Kulu and González-Ferrer 2014). Indeed, it appears that there has been almost no prior analysis of the profile of differentials across the reproductive life course of immigrants that allows us to distinguish between tempo (birth timing) and quantum (number of children born) within and between origin groups. In addition to providing an overview of differentials by age, life course variation in differentials is important because it demonstrates differences between the fertility of immigrants and natives (or other reference groups) at different stages of childbearing. For example, the analysis of cumulative (cohort) fertility differentials over the whole life course can show whether they exist at early ages, whether they diminish with age, and how they relate to differentials at the end of childbearing. As compared with cross-sectional studies of age variation, cohort analysis makes it possible to answer these questions without the need to compare people of different ages at a single point in time, thus avoiding the assumption that age-specific period rates represent a reliable picture of the trajectory of immigrant childbearing. Perhaps most importantly in the context of immigrant fertility differentials, a cohort approach allows childbearing histories prior to migration to be incorporated into the analysis. A cohort analysis of cumulative fertility can also identify differences between the tempo and quantum of fertility for different types of immigrant.

Although demographers often discuss quantum with reference to completed fertility, the term can be more generally defined as the frequency that an event occurs (e.g. number of births), and hence can be measured at any age (Bongaarts and Feeney 1998; Pressat 1985; Ryder 1980). If quantum differentials in cumulative fertility—between immigrants and natives—are estimated for a (closed) cohort at a given age, and then compared with differentials for the same cohort at a later age, then it is likely that changes in these differentials can be attributed to differences in the tempo of immigrant births, as compared with natives (and between the ages in question). For example, if there is no difference between immigrants and natives in number of children ever born at age 30, but a difference at age 40, then this change is most likely due to differences in tempo between immigrants and natives from ages 30–40. In this way, the relative variation in quantum and tempo can be contrasted, thereby highlighting differences between immigrant and native childbearing over the life course. Such comparisons therefore show the age at which immigrants are most likely to have an impact on population change (via their fertility). And they can also be used to examine the heterogeneity of quantum and tempo for different immigrant groups.

Previous research has yet to use a cohort approach to study the cumulative fertility differentials between immigrants and natives, and this appears to be equally true of research in the UK as it is elsewhere. For example, previous studies of the UK have indeed examined how immigrant birth rates vary by age as compared with those of natives (e.g. Dubuc 2012, 2016), but they have not examined cohort variation in these birth rates. In particular, the novelty of this study is that it examines how relative differences between immigrant and native fertility vary over the childbearing life course. Here, this is done by studying the childbearing trajectories of a closed cohort of women from ages 15 to 40, which can be contrasted, for example, with previous studies of age-specific fertility rates in the UK, which have not presented evidence about relative differences in childbearing trajectories, (typically due to the implicit limitations of cross-sectional data). Another contribution of this study is that it provides information about immigrant fertility in the UK for a wide range of origin groups—including women from the Caribbean, Africa, South Asia, East Asia, Europe, North America and Oceania—some of which are relatively understudied.

The difficulty of using a life course approach for the study of immigrant fertility is particularly evident from the way that immigrant fertility has been measured and analysed. Most research has analysed differentials using summary measures of fertility like the period Total Fertility Rate (TFR) (e.g. Coleman 1994; Haug et al. 2002; Ng and Nault 1997; Toulemon 2004; Toulemon and Mazuy 2004), measures that focus on fertility at a particular stage of the life course, like first birth timing (e.g. Andersson and Scott 2005; Batson 2013; Lübke 2015; Milewski 2007, 2011; Mussino and Van Raalte 2012) or completed fertility (e.g. Mayer and Riphahn 2000; Parrado 2011; Parrado and Morgan 2008; Rosenwaike 1973; Young 1991). Many studies are unable to investigate the whole reproductive life course (i.e. completed fertility profiles), either because they study samples of data that include women who have not completed childbearing (e.g. samples of women aged 15–45), or because they have no data on the childbearing of immigrants prior to arrival. Studies of life course differentials, that are not similarly constrained, can therefore be used to provide insights that are obscured in other studies, which do not analyse fertility variation over the individual life course. This is important, for example, because research suggests the period TFR may exaggerate the size of immigrant fertility differentials (e.g. Dubuc 2016; Parrado 2011; Robards and Berrington 2016; Sobotka and Lutz 2011; Toulemon 2004, 2006; Toulemon and Mazuy 2004), in particular when compared with completed fertility (Parrado 2011). One of the main reasons for this is the established link between birth timing and age at migration, such that birth rates are ‘elevated’ (relative to the native population) immediately after the age of arrival (e.g. Robards and Berrington 2016; Toulemon 2004, 2006; Toulemon and Mazuy 2004). The analysis of life course differentials by age at migration can therefore help to identify the appropriateness of different fertility measures for the analysis of immigrant fertility, and this is not only applicable to the period TFR. For example, if differentials only exist at early ages, then an analysis of first birth risks may be more appropriate than an analysis of completed fertility.

In summary then, there is a notable gap in the literature on immigrant fertility. There is a lack of research that has studied the cumulative fertility differentials using a cohort approach. And yet there is a need for this research because it enables us to understand the potential problems that arise when deploying widely used period fertility measures such as the TFR to study immigrant fertility. Comparisons of relative childbearing across the life course also have the potential to generate unique insights about distinctions between the tempo and quantum of immigrant fertility. This study responds to the gap in the literature by investigating two related questions: How do immigrant fertility differentials vary over the reproductive life course? And how does this variation over the life course compare for different groups of immigrants? The latter is particularly important given that immigrant fertility differentials have been found to vary considerably, in particular by age at migration and county of birth (e.g. Andersson 2004; Coleman 1994; Haug et al. 2002; Toulemon and Mazuy 2004). The next section provides more detail about the benefits of studying immigrant fertility differentials over the life course. The rest of this article then answers the above research questions with a case study of the life course fertility of immigrants and natives in the UK.

## Understanding Immigrant Fertility over the Life Course

### Explaining Immigrant Fertility Behaviour

In addition to describing fertility trends, studies of cumulative fertility differentials over the life course can also help researchers to explain immigrant fertility behaviour. These explanations may be based on the comparison of cumulative fertility differentials over the life course (i.e. at different ages), or the comparison of cumulative fertility differentials across groups. For example, comparisons by age make it possible to establish whether cumulative differentials at early ages are sustained until childbearing is completed. Alternatively, comparisons across groups can help researchers to gain insights about the broader determinants of fertility, for example by examining how cumulative fertility differentials vary by country of birth and age at migration in order to explore the social and cultural determinants of fertility.

Researchers have developed numerous hypotheses to explain the fertility behaviour of immigrants and why this differs from the fertility of the destination population (Coleman 1994; Goldscheider and Uhlenberg 1969; Goldstein and Goldstein 1981; Hervitz 1985; Kulu 2005; Milewski 2010; Parrado and Morgan 2008; Ritchey 1975; Zarate and de Zarate 1975). These hypotheses are too numerous to investigate in any one piece of research and are not necessarily straightforward to test, even in isolation. This is not least the case because some hypotheses imply predictions about quantum, some imply predictions about tempo, and some imply predictions about the entire childbearing profile. Nevertheless, these hypotheses will provide the background for the descriptive findings presented in this study.

In this article, we do not seek to isolate and test any particular hypotheses. The aims of this research, and its research questions, are first and foremost descriptive. Nevertheless, a comparison of life course differentials can help to narrow the potential list of explanations for the fertility of a given migrant group. It can also show which groups, and which stages of the life course, merit further investigation. As such, although it may not be possible to carry out a robust test of specific hypotheses without bespoke research designs, the analysis of life course differentials can provide an indication that some hypotheses are more plausible than others. This is particularly the case, when the analysis disaggregates migrants by origin (e.g. country of birth) and age at migration. It is for this reason that this section describes the hypotheses that are used to explain immigrant fertility (which are also described in detail in Tables 2 and 3 of the ‘Appendix’, alongside their predictions). In doing so, we aim to guide the reader’s interpretation, and the limits of this interpretation, with respect to our descriptive results.

For example, *cultural entrenchment* predicts that the fertility of certain immigrant groups will be influenced by their lack of exposure to destination culture (e.g. destination fertility norms) (Abbasi-Shavazi and McDonald 2000; Forste and Tienda 1996; Milewski 2010). Given this prediction, it is more difficult to argue for cultural entrenchment in the absence of differentials, especially if the focus is on immigrant origins that have different fertility norms from the destination. In contrast to cultural entrenchment, *childhood socialisation* predicts that migrant fertility depends upon the fertility preferences that migrants are exposed to in childhood (Hervitz 1985). This implies that the fertility of ‘adult migrants’, who migrate after the end of childhood, will be similar to the fertility of their origin country. As such, an absence of fertility differentials is usually expected only for child migrants, who arrive in a destination before the end of childhood (and before childbearing has begun). Of course, socialisation will depend upon the precise interaction between migration background and childhood context, which will determine whether mainstream norms have a prevailing (socialising) impact on childbearing behaviour. Nevertheless, as has been argued elsewhere (e.g. Milewski 2010), an absence of differentials for the descendants of immigrants provides some indicative evidence in support of childhood socialisation.

The reason that this evidence is only indicative is because of the likelihood that there are alternative explanations for a lack of child migrant differentials. There are several hypotheses that predict a link between the timing of migration and the timing of fertility for adult migrants. These include that fertility is disrupted by migration (*disruption*) and that fertility is elevated after migration because migration is linked to partnership behaviour (*family formation*) (Goldstein and Goldstein 1983; Milewski 2010). Although these hypotheses are hard to assess without reference to the population in an immigrant’s country of origin (Hoem and Kreyenfeld 2006a, b), they do not apply to child migrants. As such, in addition to childhood socialisation, a lack of differentials for child migrants might be explained by the fact that, unlike adult migrants, the timing of their migration and fertility are not interlinked. In other words, if the timing of migration is the sole explanation for adult migrant differentials, then we would expect an absence of differentials for child migrants, irrespective of socialisation.

The importance of migration timing for adult migrants suggests that the analysis of differentials by age at migration can help inform explanations for immigrant fertility, especially if it allows child and adult migrants to be distinguished. Age at migration is also linked to ‘exposure to destination’, which can be measured by duration of residence (age minus age at migration). Convergence over exposure to destination can therefore be evaluated by comparing how life course differentials vary by age at migration. Similar to research on ethnic fertility differentials, this analysis can be used to investigate exposure to destination as a determinant of fertility.

Again, caution is required when analysing differentials by exposure. *Adaptation* predicts immigrant fertility convergence over the life course (after arrival) due to exposure to destination norms and institutions, or due to adaptation to new socio-economic circumstances (Andersson and Scott 2005; Harbison and Weishaar 1981; Milewski 2010). This suggests that adaptation might be supported by profiles that show large fertility differentials immediately after migration (i.e. *elevated fertility*), as long as these profiles then gradually disappear with age (and especially if profiles at later ages are very different at origin or we can control for the selectivity of immigrants). However, adaptation is hard to assess for adult migrants because elevated fertility after migration might have a range of alternative explanations (Hoem and Nedoluzhko 2016).

These include the possibility that certain types of immigrants are selected from the origin population (*selection*) or that women’s propensity to migrate is increased if they do not have a child (*reverse causality*) (Harbison and Weishaar 1981; Toulemon 2006). As a third alternative, immigrants may delay childbearing until after migration, as a form of disruption of childbearing and *anticipation* of their migration (Milewski 2010). Despite the difficulties of isolating any single explanation, it is possible to provide some evidence about adaptation by exploring the differentials for child migrants. Slightly different from childhood socialisation, one expectation of adaptation is that child migrants have differentials that become smaller as they approach the end of their childbearing. This is because they will have a longer time to adapt to the destination norm for completed fertility than the norm for early childbearing.

### The Benefits of a Study of Cumulative Fertility Differentials

Given the potential benefits of a study of immigrant’s cumulative fertility differentials, it is perhaps surprising that such studies appear to be absent from the literature. With respect to national populations as a whole (in absence of any differentiation between immigrants and natives), there have been many researchers who have used a cohort approach to study fertility (notably, see: Frejka and Calot 2001; Frejka and Sardon 2007). There are some studies that have analysed the completed and partially completed fertility profiles of immigrants (e.g. Alders 2000; Bagavos et al. 2007; Fokkema et al. 2008; Friedlander and Goldscheider 1978; Garssen and Nicolaas 2008). However, there do not appear to be any studies that have attempted to calculate, analyse, and compare cumulative fertility differentials—versus natives—over the entire reproductive life course for different immigrant origin groups.

As discussed in the introduction, most of what we know about immigrant fertility differentials is either based on period measures of fertility (like the TFR), or on the examination of part of the reproductive life course, (e.g. first birth rates). There has been limited prior analysis of the profile of differentials across the reproductive life course of immigrants, and no previous research that allows us to distinguish between tempo and quantum variation within and between immigrant origin groups. This is an important gap because, unlike other approaches, such an analysis makes it possible to understand distinctions between the tempo and quantum of immigrant fertility. This study therefore aims to redress this gap by describing how immigrant fertility differentials vary over the reproductive life course, and how this life course variation is different for different groups of immigrants. In order to do this, it carries out an empirical study of the UK.

## Context and Data

### The UK Context

There are several reasons why the UK is an excellent case for the study of immigrant fertility, especially in Europe. Compared to most other high income countries, the UK has a long history of immigration from a diverse range of origins (Coleman et al. 2002; Rendall and Salt 2005; Walvin 1984). The existence of sizeable groups of older migrants (ONS 2012b; Rendall and Ball 2004; Rendall and Salt 2005), means that it has a large population of immigrant women who have completed their fertility. Importantly, the UK also has data that allow life course differentials to be studied for different immigrant groups, including data on childbearing prior to immigration.

Over the last few decades, the UK is among the European countries that have experienced increases in the size of their foreign-born populations (Coleman 2009; Haug et al. 2002). Accompanying this trend, there has been a keen policy interest in the fertility behaviour of migrant fertility, including as part of a broader debate about the impact and integration of new waves of immigrants (Allen and Warrell 2013; BBC 2013; Easton 2012; Sedghi 2014). As with many other European countries, there is some evidence of immigrant fertility differentials in the UK (Coleman 1994; Dormon 2014; Dubuc 2012; Iliffe 1978; Murphy 1995; Robards and Berrington 2016; Sigle-Rushton 2008; Sobotka 2008; Tromans et al. 2007; Waller et al. 2014; Zumpe et al. 2012). However, there is limited knowledge about these differentials because they have not been analysed over the reproductive life course. To be more specific, there has been some analysis of variation in period fertility by age, for example using age-specific fertility rates (ASFRs) (e.g. Dubuc 2016), as well as analysis of parity-specific fertility rates (e.g. Kulu and Hannemann 2016; Kulu et al. 2017). However, none of this analysis has focused on specific cohorts of women to examine the profile of differentials across the reproductive life course. Likewise, there is no published UK research that allows us to distinguish between the tempo and quantum of fertility within and between immigrant origin groups. The lack of research on this topic no doubt reflects the fact that it places substantial requirements on the need for longitudinal data covering the reproductive life course (i.e. for women aged 40+), with a large enough sample to study separate immigrant origin groups.

The UK is comprised of four constituent countries, which are: England, Wales, Scotland, and Northern Ireland. The history of migration to the UK from different origins is considerable (e.g. Coleman et al. 2002; Daley 1998; Foner 2009; Hornsby-Smith and Dale 1988; Horsfield 2005; Murphy 1995; Peach 2006; Rendall and Ball 2004; Rendall and Salt 2005; Walvin 1984). Historically, the largest group of immigrants to the UK have come from Ireland, but since 2001 they have been replaced by Indians as the largest foreign-born group (ONS 2012b). Indian migration began in earnest in the late 1960s and early 1970s, and this was closely followed by significant inflows of migrants from Pakistan around the mid-1970s, and then migration from Bangladesh which gathered pace at the end of the 1970s and the beginning of the 1980s (Coleman et al. 2002). In contrast to these South Asian origins, immigration from the Caribbean was at its peak in the 1950s and 1960s, and then fell considerably after the Commonwealth Immigrants Act introduced restrictions in 1962 (Foner 2009). Nevertheless, much family reunification occurred after the Act, which led to continued immigration of Caribbean women throughout the 1960s. Of the other origins and origin groups that are analysed here, immigrants from the ‘Old Commonwealth’ countries (New Zealand, Australia and Canada) have a considerable history of settlement in the UK. This can be contrasted with Eastern European and African immigrants who have only migrated in significant numbers more recently, albeit from a diverse range of origin countries (Daley 1998; ONS 2013).

### Data

This research uses data from the first wave of Understanding Society (UKHLS), which are representative of the UK population, and includes responses for more than 50,000 adults who were surveyed between 2009 and 2011 (University of Essex 2011). For the analysis that follows, the analytical sample includes a total of 11,096 adults, all of whom are women aged between 40 and 70 (i.e. born between 1941 and 1971), who were not surveyed by proxy, and who migrated before they were aged 36 (if they are foreign-born). The latter restriction ensures that all women have been resident in the UK for at least 5 years before their completed fertility is measured at age 40. Tables 4 and 5 in the ‘Appendix’ provide descriptive statistics for the analytical sample. Importantly, the sample size is sufficiently large for specific country of birth groups to be separately identified, and to facilitate the analysis of three groups by age at migration: under 16, 16–25, and 26–35.

For the purposes of this research, the number of children ever born at age 40 serves as an indicator of completed fertility at the end of a woman’s reproductive life span. Although this clearly ignores a small number of births that occur after this age, on average this is only equivalent to a mean difference of 0.03–0.05 children (see Table 5 in the ‘Appendix’). By choosing 70 as the upper age limit, we hope to avoid bias that may be a result of differential mortality between immigrants and natives (although this bias depends on differences in mortality by fertility history, which do not appear to have been studied in the UK)

When comparing our analysis with statistics based on other sources, such as registered births in England and Wales, it is important to acknowledge differences in the populations that they represent. Registered births are recorded at the time of birth, whereas the UKHLS sample represents the fertility of women who are alive and resident in the UK at the time of survey. There is no research on the UK that can offer guidance whether this is likely to impact the results. However, research on Swedish data suggests that mortality and migration may make little difference to aggregate estimates of fertility; however, they may have more of an influence when comparing natives with the more mobile migrant population (Andersson and Sobolev 2013).

Nevertheless, the UKHLS fertility histories were checked using a comparison of two different parts of the UKHLS. Histories were initially obtained using information on non-resident children from the woman’s birth history and information on resident children from the woman’s household questionnaire. These results were then compared against an alternative calculation using the birth history questions for both resident and non-resident children. Comparisons suggested there was relatively little difference between the two calculations, although the preferred method (using the household questionnaire for resident children) gave slightly higher estimates of average fertility (see Table 4 in the ‘Appendix’). Information from the household relationship matrix was then used to check for errors, and as a result around 100 cases were corrected for inconsistencies.

## Method

### Research Design

The analysis compares fertility profiles of immigrants and natives longitudinally, such that the (cumulative) number of children born to immigrants is compared with the number of children born to natives at the same age. This comparison is presented as a ratio, which represents the immigrant fertility differential for a given group of immigrants at a given age.

It is important to note that all births up to age 40, before and after migration, are known for all women in the sample, so for the purposes of this analysis fertility is complete. Given that births are a rare event over the entire life course, comparisons are made by single years of age. This is important because comparisons of differentials between groups are likely to be highly sensitive to even small changes in childbearing, especially since the average completed fertility of both immigrants and natives is not much more than two children per woman.

Comparing the unweighted counts of foreign-born and UK-born women in the analytical sample, they appear to have similar distributions across several covariates that are relevant for the study of fertility, including education and partnership. However, covariates are not used in this analysis, not least because most relevant covariates (like partnership and education) are simultaneous to the fertility process (i.e. endogenous, hence acting as both causes and effects), and their inclusion (given our research questions) would therefore bias the findings and make them very difficult to interpret.

### Statistical Approach

The statistical analysis uses count regression models to estimate children ever born at each age (Agresti 2002). These models have been used previously by research on migrant fertility (Adserà and Ferrer 2014a; Adserà et al. 2012; Mayer and Riphahn 2000). In each stage of the analysis, a set of models are estimated at a range of ages, from 20 to 40, using children ever born (at a given age) as the response variable. As such, each stage begins by estimating a model for the entire analytical sample based on number of children born at age 20, and then repeats this analysis for the same sample at age 21, 22, 23… (etc.), up to age 40. The first stage of the analysis is to estimate a series of models comparing foreign-born and native-born women. In subsequent stages, the analysis is repeated, but using different categorisations for foreign-born women. Natives are always grouped together and are used as the reference group throughout (including the second generation).

The models are defined as follows: Let $Y_{ij}$ denote the number of children ever born for individual *i* at age *j*. As the only explanatory variable, $\varvec{G}_{j}$ is an indicator variable for immigrant group, which is defined in the same way at each stage of the analysis (i.e. for each set of models that are estimated at each age from 20 to 40), but varies at different stages according to the migrant groups that are investigated. As such, $\varvec{G}_{j}$ can indicate nativity (i.e. whether native-born or foreign-born), country of birth group, age at migration group, or a group that indicates both country of birth and age at migration. The outcome is then modelled such that $Y_{ij}$ follows a Poisson distribution with expected value:

$$E\left( {Y_{ij} } \right) = { \exp }\left( {\alpha_{j} + \beta_{j} \varvec{G}_{i} } \right),$$

for *i* = 1,…, *n*, estimated separately for each age *j* = 20,…, 40, where $\beta_{j}$ is a vector of coefficients for $\varvec{G}_{j}$ that vary by age and immigrant group. At age *j*, a risk ratio for each migrant group, compared to the reference group of UK-born women, is therefore defined as: ${\text{IRR}}_{j} = \exp \left( {\beta_{j} } \right)$. These are referred to here as immigrant fertility differentials. The models are estimated so that a ratio above 1.0 is a ‘positive’ differential, indicating that immigrants have more births than natives on average, and a ratio below 1.0 is a ‘negative’ differential, indicating immigrants have fewer births on average.

All regressions were estimated using the *svy* command in Stata version 11, to account for the complex survey design of the UKHLS (StataCorp 2009). This means that the results are adjusted for unit non-response, as well as the fact that immigrants, or more specifically ethnic minority groups, are oversampled in the survey. For comparison, negative binomial models were also estimated, and the estimates and standard errors were virtually identical.

## Results

### Average Differences Across the Fertility Schedule

Before examining differentials over the life course for different origin groups, it is useful to look at the general fertility trends. Typically, this general trend is either estimated using period TFRs or cohort fertility, and Table 1 compares both of these measures. As with previously published comparisons, we compare the TFR for a given year with completed fertility for cohorts of women who were born 30 years earlier (e.g. ONS 2011; Smallwood 2002; Sobotka 2004). This approximates the mean age at birth of the cohort (the standardised mean age of mother was 29.7 in the UK in 2011: ONS 2016) and is a conventional approach that helps to facilitate a comparison between the two measures. In Table 1, but not elsewhere in this article, completed fertility is calculated for England and Wales, rather than the UK. This allows an equivalent comparison with published TFRs, although this change in coverage makes little difference (see Tromans et al. 2007; Table 1, for the difference it makes to TFRs), largely because England and Wales accounts for 89% of the UK population by size (ONS 2014).

**Table 1:** Period total fertility rate (for women aged 15–45) versus completed family size (for women aged 40 plus, birth cohorts + 30 years)

England and Wales	1981	1986^a^	1991	1996	2001	2006	2011
TFR							
UK-born	1.7	1.7	1.8	1.7	1.6	1.8	1.9
Foreign-born	2.5	2.5	2.4	2.5	2.2	2.4	2.3
Differential	0.8	0.8	0.6	0.8	0.6	0.6	0.4
Completed fertility (+ 30 years)							
UK-born	2.1	2.0	2.1	2.0	2.1		
Foreign-born	2.0	2.4	2.1	2.1	2.3		
Differential	− 0.1	0.4	0.0	0.1	0.2		

*Source*: Office for National Statistics and UKHLS data (author’s analysis). Coverage: England and Wales (i.e. the UK excluding Scotland and Northern Ireland)

Completed fertility results are shown for birth cohorts plus 30 years, to facilitate comparison with the period TFRs, where 30 years is chosen as the approximate mean age of childbearing

^a^Result for 1985

Table 1 shows that there is a smaller immigrant fertility differential for completed fertility than for the period TFR. Although foreign-born women have a TFR that is almost always more than half a child larger than UK-born women, the completed fertility differential is far smaller, and even equals zero for the 1991 comparison. Table 1 therefore warns against using the foreign-born TFR in order to infer completed fertility differences compared with natives. It is interesting to note that, based on Table 1 alone, researchers might assume that there are no differences between the fertility of immigrants and natives. In the analysis that follows, the results show that this is not the case, and that cumulative fertility differentials vary considerably, both over the reproductive life course, and for different migrant groups.

### Differentials Across the Fertility Schedule

**Fig. 1:**
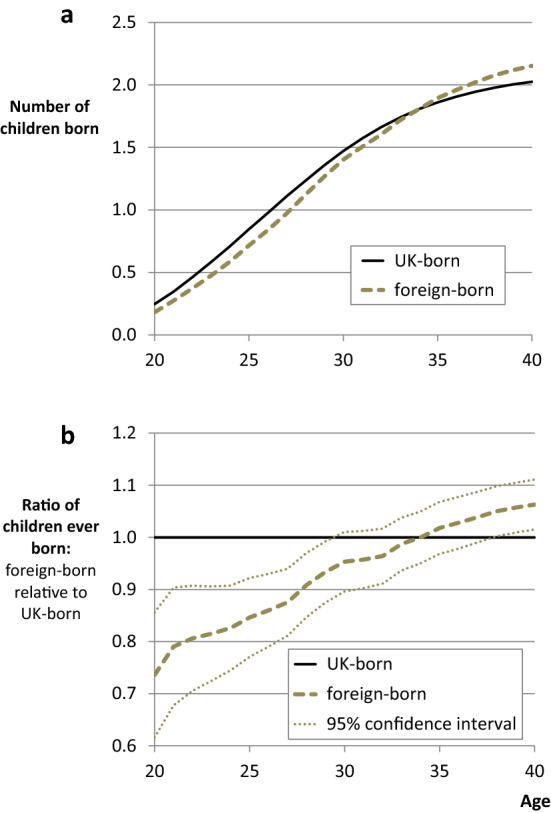
Fertility profiles of children ever born by nativity. Note: **a** and **b** report the results from 21 separate Poisson regression models (one for each age). The analytical sample is the same for each model. As such, the analysis compares the same groups of immigrants and natives, born between 1942 and 1971, at different ages. The ratio of children ever born (all births up to a given age) is obtained from the modelled IRR of foreign-born women relative to UK-born women. UK-born women therefore have a ratio of 1.0 at all ages. *Source*: UKHLS data (author’s analysis)

Despite the fact that complete fertility differentials are fairly small, Fig. 1 shows that immigrants have (on average) given birth to significantly lower numbers of children than natives at early childbearing ages. At age 20, the average number of children born by immigrant women is lower than UK-born women by a factor of 0.75. This differential becomes smaller as age increases, but it is not until the middle of their reproductive life course that immigrants catch-up towards the native norm.

Figure 1 therefore demonstrates one of the advantages of analysing fertility differentials over the life course. The charts in the rest of this article have the same *y*-axis as Fig. 1b and show the profile of differentials, but in this case Fig. 1a shows the actual fertility profiles that are used to calculate these differentials. Given that the sample remains the same at each age, the relationship between differentials over the life course can be compared directly. It is worth noting that differentials are expected to be slightly more sensitive at early ages because levels of childbearing are smaller. Nevertheless, based on the variation in differentials shown in Fig. 1b, it seems reasonable to ask which immigrant groups are responsible for the shape of this profile, and how much heterogeneity lies behind it.

### Variation by Country of Birth

**Fig. 2:**
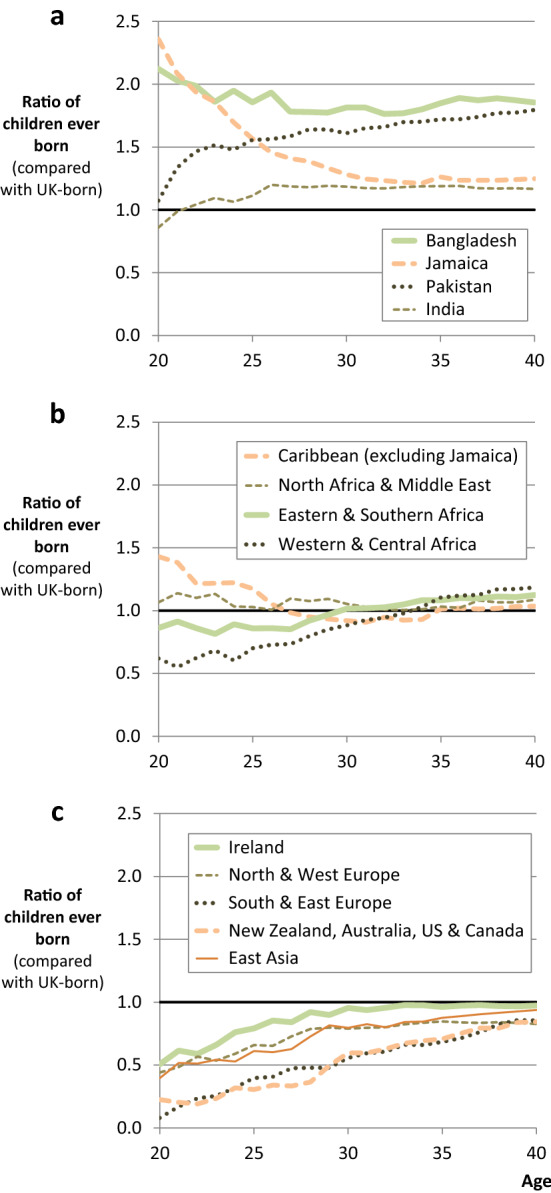
Differentials by country of birth. Note: **a** shows groups with a higher completed fertility than natives, **b** shows groups with a similar completed fertility to natives, and **c** shows groups with a lower completed fertility than natives. Results for **a**–**c** obtained from a series of Poisson regression models, where the outcome is children ever born (all births up to a given age). The analytical sample is the same for each model. As such, the analysis compares the same groups of immigrants and natives, born between 1942 and 1971, at different ages. The reference category for these differentials is UK-born women (who effectively have a differential of 1.0 at all ages). *Source*: UKHLS data (author’s analysis)

Figure 2 shows that there is considerable variation in the profile of immigrant fertility differentials by country of birth (i.e. immigrant origin). In this and the following figures, we do not report estimates of uncertainty in order to focus on the general patterns with respect to the profile and magnitude of quantum differentials. In doing so, we note that many of the results are significant, and the analysis is not underpowered. The confidence intervals and associated *p* values vary by country of origin, but in general *p* values are broadly proportional to the proximity of an IRR to 1.0 (the reference value for UK natives). For example, the IRRs for Indians in Fig. 2 are close to 1.0 at ages under 25, and their fertility is not significantly different from natives at the 1% level. However, at ages 30 and over, their IRRs are larger and further from 1.0, and their fertility is significantly different from natives at the 1% level. This can be compared to the IRRs for Bangladeshis, which are above 1.75 and significant at the 1% level at every age from 20 to 40.

Based on a comparison of origins at age 40, we can identify the origin groups that have the largest completed fertility differentials. These immigrants—Jamaicans and South Asians (Indians, Pakistanis, and Bangladeshis)—therefore make the largest eventual contribution to population size in terms of their fertility rates (i.e. conditional on their absolute numbers in the population). However, it is also apparent that they have very different profiles, such that their fertility impacts population dynamics in different ways at different ages. For example, compared with Pakistani women, Bangladeshi women have very similar completed fertility, but far higher differentials at ages under 30. The quantum of fertility at age 20 is almost the same as natives for Pakistani-born women, whereas births to Bangladeshi-born women are (on average) much earlier than both Pakistanis and natives. A similar comparison can be made between women from India and Jamaica, who have very similar completed fertility, but very different profiles, such that the childbearing of Jamaicans begins much earlier (with a significant differential of more than 2.3 at age 20). Overall, these patterns suggest that while Bangladeshis and Pakistanis may eventually make the greatest relative contribution to population size (i.e. net of their absolute numbers), Bangladeshis and Jamaicans (in these cohorts) were more likely to have an earlier (relative) impact on population change via their (early) childbearing.

For some origin groups, the profile of differentials is more stable over the life course. This is the case for African and Middle Eastern origins, as well as women who were born in the Caribbean outside Jamaica. However, even among these aggregated groups, there is evidence of differences in birth timing. Differences in timing are also evident for origin groups that have lower levels of completed fertility to natives (i.e. negative differentials). For example, Southern and Eastern European migrants are much less likely to have children at young ages, as compared with UK natives. At age 20, their average number of children born is lower than UK-born women by a factor of 0.08 (95% CI 0.01; 0.56). This compares with a factor of 0.85 at age 40 (95% CI 0.71; 1.02), which although still below 1.0, is much more similar to the native norm.

For Southern and Eastern European migrants, this pattern may reflect the (relatively lower) fertility of their origin countries, which is not only the case for recent estimates of period fertility, but also for cohort fertility for the cohorts that are studied here (Frejka and Calot 2001; Frejka 2008). For example, there has been a steep decline in second birth total cohort fertility rates in for women born after the 1950s in Southern and Eastern Europe (Frejka and Sardon 2007) and an increase in childlessness (Frejka 2008). Although there has been a somewhat similar broad pattern of postponement for the same birth cohorts of women in the UK—i.e. the native group that are used as the comparison—the decline in both period and cohort fertility has been less marked in the UK (for example, see: Frejka and Calot 2001).

However, the pattern that we observe for immigrants from Southern and Eastern Europe is not exclusive to these origins and is almost the same as the profile of differentials for women from the USA and ‘Old Commonwealth’ countries (New Zealand, Australia, and Canada), which suggests that this pattern of childbearing is not necessarily associated with origin fertility norms. Alternative explanations, which could be true for all origin groups that exhibit negative differentials, is that this pattern of differentials may be driven by disruption, or by the selection of migrants who are more likely to postpone childbearing and end their reproductive lives with fewer children than UK-born natives.

### Age at Migration

As discussed, age at migration is likely to be linked to changing patterns of immigrant fertility differentials. The majority of immigrants arrive as adults, which means that they migrate after the start of their reproductive years. For example, more than two-thirds of immigrants who were born outside the UK and resident in England and Wales in 2011 had an age at arrival between 15 and 44 (ONS 2012a).

One of the difficulties for assessing fertility differentials for adult migrants is the fact that the timing of their childbearing and their immigration are likely to be associated with each other (Andersson 2004; Hoem and Nedoluzhko 2014; Milewski 2007; Robards 2012; Toulemon and Mazuy 2004). This can be contrasted with child migrants, whose fertility is much less likely to be associated with the timing of their migration, not least because they usually arrive before their fertile years begin (e.g. ONS 2012a). This implies that the differentials for child migrants may provide indicative evidence about the patterns of adult migrant fertility that would have occurred if their migration had occurred earlier. Although as noted already, there are a range of potential explanations for any differences, including selection, adaptation, and childhood socialisation.

**Fig. 3:**
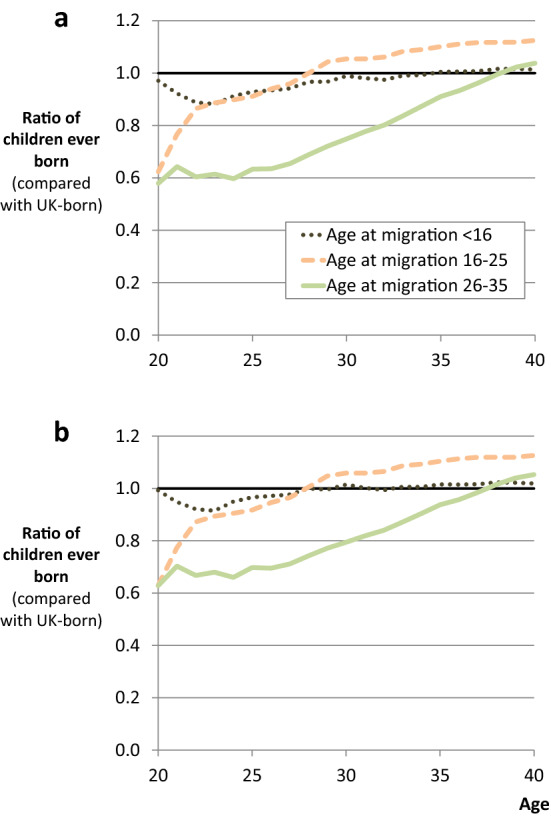
Differentials in cumulative fertility by age at migration, female cohorts 1942–1971. Note: results are obtained from a series of Poisson regression models, where the outcome is children ever born (all births up to a given age). The analytical sample is the same for each model. As such, the analysis compares the same groups of immigrants and natives, born between 1942 and 1971, at different ages. The reference category for these differentials is UK-born women (who effectively have a differential of 1.0 at all ages). *Source*: UKHLS data (author’s analysis). Each of the two plots represents a different model specification. The first is without controls for birth cohort. The second is with controls for birth cohort group (1942–1951, 1952–1961, and 1962–1971)

As shown in Fig. 3, there is less variation in differentials for child migrants, as compared with adult migrants in the UK. Not only is there a lack of variation in differentials over the life course (i.e. the profile is horizontal), but there is almost no evidence of differentials at any age. This is a new finding for the UK and suggests that child migrants who are resident in the UK have a very similar fertility profile, on average, to UK-born women.

For adult migrants, it is evident that the profile of their differentials depends upon their age at migration. As with all these results, the differentials in Fig. 3 include births before and after migration, and from this we can see that immigrants who arrive in the UK aged 16–25 have fewer children than natives at age 20 by a factor of 0.62 (95% CI 0.48; 0.81). However, by age 25, after all these women have arrived in the UK, this differential is 0.91 (95% CI 0.80; 1.04). And by age 29, their average differential has switched from negative to positive (i.e. the ratio has changed from below 1.0 to above). Although further refinement would be required to consider whether births occurred just before, or just after migration, this seems to confirm that there is a strong relationship between the timing of migration and childbirth (for example as shown for the UK by Robards and Berrington 2016). In addition, despite the lower fertility of these migrants at early ages, by age 40 they have significantly more children than natives, on average by a factor of 1.12 (95% CI 1.05; 1.20). This profile can be compared with adult migrants who arrived when aged from 26 to 35. With an average number of children born that is lower than natives at age 20 and age 25, this group exhibits a similar pattern of low fertility prior to migration. Importantly, they also ‘catch-up’ with native fertility levels by age 40, implying a period of elevated fertility either shortly before or shortly after migration. Moreover, the results suggest that age at arrival is a much stronger predictor of differentials at early childbearing ages than it is at the end of childbearing (i.e. for completed fertility).

**Fig. 4:**
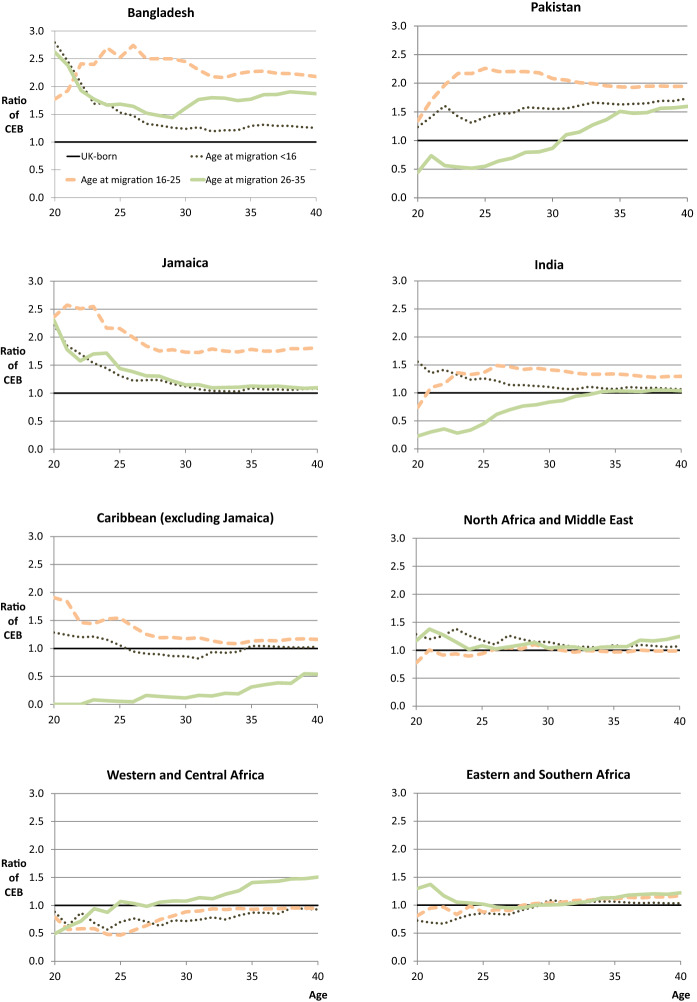

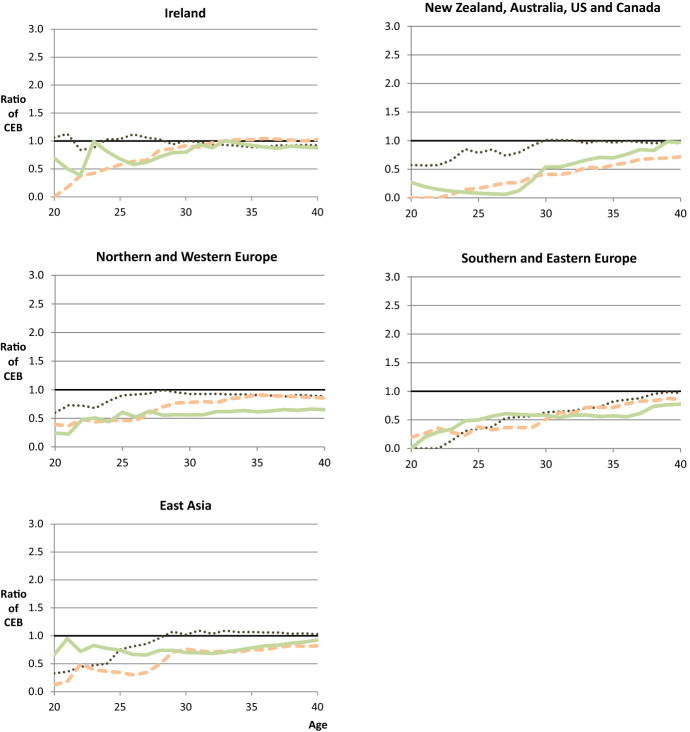
Differentials in cumulative fertility by age at migration and country of birth, female cohorts 1942–1971, controlling for birth cohort. Note: results are obtained from a series of Poisson regression models, where the outcome is children ever born (all births up to a given age). All models control for birth cohort group (1942–1951, 1952–1961, and 1962–1971). The analytical sample is the same for each model. As such, the analysis compares the same groups of immigrants and natives, born between 1942 and 1971, at different ages. The reference category for these differentials is UK-born women (who effectively have a differential of 1.0 at all ages). *Source*: UKHLS data (author’s analysis)

Figure 3 also compares the results of two different specifications: one with and one without controls for birth cohort. After controlling for birth cohort, the results for those arriving aged 26–35 change very slightly, with relative differences from the UK-born becoming marginally smaller at early childbearing ages. However, the inclusion of birth cohort makes very little difference to the results, and no difference to the above interpretation. (And the same is true below for the analysis in Fig. 4, so we only present results controlling for birth cohort group.)

Taken as a whole, these results demonstrate the patterns of tempo-variation by age at migration, which may be the cause of tempo-distortion when analysing migrant fertility using samples of women including those who have yet to complete childbearing. They also show the importance of accounting for age at migration when analysing fertility differentials, especially at early ages. Given this variation by age at migration, and the variation observed by country of birth, a useful next step is to see how these two characteristics interact.

### The Relationship Between Country of Birth and Age at Migration

The results in Fig. 4 show that there is considerable heterogeneity in life course differentials when analysed by country of birth and age at migration. In general, the child migrants of most origin groups tend to have smaller differentials than adult migrants and exhibit fertility behaviour that is closer to natives at all stages of the life course. This evidence could support several explanations, including childhood socialisation and adaptation. When making this interpretation, it is important to note that the fertility of child migrants is not interrupted by their immigration and is far less likely to be confounded by selection (Adserà et al. 2012).

Unlike adult immigrants, the life course differentials in Fig. 4 for child migrants cannot be explained by hypotheses that predict an interrelationship between migration and fertility (like disruption). As such, they are much more likely to be explained by (a combination of) childhood socialisation and adaptation after arrival. The results for child migrants from Pakistan are notable because, unlike other origins, they become more different from UK natives over the life course (rather than more similar). This may be due to childhood socialisation and a lack of adaptation. The evidence in support of socialisation is that early childbearing appears less common among those arriving before age 16, in comparison with those who spent their whole childhood in Pakistan and arrived aged 16–25. The evidence in support of a lack of adaptation is that irrespective of spending some of their childhood in the UK, their differentials become larger over the life course. Of course, these interpretations are tentative, for the reasons given earlier. For example, childhood socialisation will depend upon the precise interaction between migration background and childhood context, and selective migration may produce fertility behaviour that looks like adaptation.

The results for adult immigrants are less easy to generalise. Immigrants from South Asia or the Caribbean who arrive aged 16–25 all have a noticeably higher fertility than natives at some point during the life course, but the profiles of differentials vary considerably, including the age at which differentials are highest. For example, Bangladeshis who arrived 16–25 have higher fertility than natives at all ages, but this is much less the case before migration (i.e. at ages < 25). A similar pattern is observed for Pakistanis, and to a lesser extent Indians. It is likely that these patterns of lower pre-migration differentials are due to a combination of the postponement of childbearing until after migration (e.g. for women who intend to migrate), alongside the lower likelihood that women will migrate if they already have children (i.e. reverse causality or selection). However, this pattern is not universal and cannot be used to explain the age profile of differentials for Bangladeshi women who migrate after age 25.

These patterns of lower pre-migration differentials are not surprising given previous research, and when combined with evidence of higher differentials after migration, they go some way towards explaining the established finding of elevated fertility soon after migration for adult migrants (Andersson 2004; Hoem and Nedoluzhko 2014; Milewski 2007; Parrado 2011; Robards 2012; Toulemon 2004, 2006; Toulemon and Mazuy 2004). It is interesting to note that similar profiles (of higher differentials at later ages) are evident for adult immigrants from many origins, not only those arriving aged 16–25, but also for many arriving aged 26–35. Notable exceptions to this pattern are those arriving aged 26–35 from Bangladesh and Jamaica. In this case, fertility appears be elevated, relative to UK natives, at early childbearing ages.

Taken as a whole, the results in Fig. 4 demonstrate that the relationship between migration and fertility is not necessarily consistent, and most certainly varies across immigrant origin groups in the UK. Some groups, such as Bangladeshis and Jamaicans, have higher fertility than UK natives prior to arrival, while many other groups do not. One possible explanation for this result relates to the higher prevalence of marriage at young adult ages among Bangladeshis as compared to the UK-born population, which may in turn be linked to marriage migration or family reunification for a number of women (Berrington 1994). Similarly, although marriage is not more prevalent among immigrants from the Caribbean, there is a higher prevalence of cohabitation and childbearing prior to marriage (Berrington 1994, 2018).

When interpreting these profiles of differentials, it is noteworthy that some groups are outliers, while others follow the (broadly) common trends. This has implications for future research, including whether the period TFR is likely to be distorted by tempo-variation, in particular by elevated fertility after arrival for adult immigrants. It is well known that comparisons between groups using the period TFR can be distorted by differences in their timing of births (Ní Bhrolcháin 2011). This issue is particularly problematic for studies of migrant fertility differentials, where the timing of migrant births is known to relate to the timing of migration (e.g. Murphy 1995; Toulemon and Mazuy 2004). In addition (unless it is adjusted), the period TFR only considers births that occur after arrival in the destination (Toulemon 2004). If immigrant birth risks are elevated after arrival, as is commonly observed, and the unadjusted period TFR is used, as is often the case, then this may lead to an overestimate of immigrant fertility differentials (Parrado 2011; Toulemon 2004, 2006; Toulemon and Mazuy 2004). Given the evidence of elevated fertility for many of the origins studied here, our results suggest that pre-migration history should be taken into account when calculating immigrant TFRs, at least for these origins (as suggested by Toulemon 2006). On the other hand, for origins where differentials are fairly stable over the life course, and do not depend upon age at migration, then it may be more appropriate to use the TFR as a proxy measure for fertility quantum. For example, this appears to be the case for immigrants from North Africa and the Middle East.

Despite the general pattern of child migrant differentials being smaller than those of adult migrants across the life course, there are some origins that diverge from this pattern. Child migrants from Jamaica, Bangladesh, and India have high differentials at young ages, suggesting an earlier timing of births compared with natives, and an absence of socialisation (which predicts a lack of differentials for child migrants—although actual socialisation will depend upon the interaction between migration background and childhood context). Moreover, this suggests that if adaptation does occur, then it is more likely to occur towards the end of childbearing, presumably because there is less time to adapt at the beginning of the life course.

By contrast, Pakistani child migrants show a very different pattern from these origins. The fact that they have almost no differential at early ages, but that their differential steadily increases with age, suggests that their relative contribution to population growth (net of their absolute numbers in the population) will be very different from other child migrant groups. This also implies that there is a different explanation for their differentials. As outlined in the introduction, most hypotheses, except cultural entrenchment, predict that the fertility of child migrants should be the same as, or converge towards native fertility (see also Tables 2, 3 in the ‘Appendix’). On the other hand, cultural entrenchment predicts that differential fertility may be sustained for child migrants due to exposure to origin subcultures (Abbasi-Shavazi and McDonald 2000; Forste and Tienda 1996; Milewski 2010). As such, the most likely explanation for the differentials of Pakistani child migrants, which are largest at the end of their reproductive life course, may therefore be cultural entrenchment. That said, as with evidence of adaptation, this result could also be explained by socio-economic ‘entrenchment’, rather than, or additional to ‘culture’.

For adult immigrants from Pakistan, the profile of differentials has a similar shape to that of Indians, although levels of completed fertility are much higher relative to natives. This result may be indicative of preferences for larger families, although there is evidence to suggest that structural factors may be more important for explaining differences between South Asians. Evidence based on ethnicity suggests that Indian women are more likely than Pakistani or Bangladeshi women to postpone marriage and childbearing, in part because they spend longer in full time education and are more likely to be employed (Berrington 1994, 2018; Dale et al. 2002). Moreover, for many Pakistani first- and second-generation women, early childbearing may be a strategic choice, determined by a ‘complex interplay of relationships between individuals, couples, and wider families’ (Hampshire et al. 2012, p.39). The importance of factors beyond the individual is also suggested by research on residential segregation, which has been shown to be associated with completed fertility for both childhood immigrants and second-generation women from Pakistan and Bangladesh (Wilson and Kuha 2017). Further research would be required, however, to tease apart the differences between these two origin groups.

In the analysis of country of birth only (Fig. 2), Jamaicans and Bangladeshis have differentials that indicate earlier childbearing than UK-born natives. Figure 4 shows that this behaviour is driven by different types of immigrants. For Bangladeshi women, it is those who migrate early or late in their life course (as children or aged 26–35) who are most likely to have earlier births. Whereas for Jamaicans, it is those who migrate aged 16–25. In fact, this is the only group of Jamaicans who have a significant fertility differential at age 40, thereby indicating that these are the Jamaican immigrants who had the largest impact on population change.

For African origins, it is interesting to note that differentials are quite similar across the groups that are analysed here. Differentials are small or seemingly non-existent at any age at migration for immigrants from North Africa and the Middle East, and the same is true for those from East and Southern Africa, (with the exception of child migrants in the early stages of childbearing). Of all the African groups, only West and Central Africa demonstrates a lot of variation by age at migration, with the most distinct pattern being for those arriving aged 26–35.

For the remaining origin groups, who all have lower completed fertility than natives on average, there are many similarities in the patterns of differentials by age at migration. Irrespective of age at migration, adult migrants in these groups generally display significantly lower numbers of children born at early childbearing ages, although this difference becomes smaller with age. This can be contrasted with the profile of differentials for child migrants from these groups, which are much closer to the average for natives. The exception is migrants from South and East Europe, where child migrants exhibit the same profile as adult migrants. These results suggest that the timing of migration for South and East European immigrants makes very little difference to their fertility. As noted in previous research, studies of migrant fertility often ignore the fertility of immigrants from origins that have lower fertility than the destination (Castro-Martín and Cortina 2015). However, as shown here, immigrants from low fertility origins may be a particularly interesting group for further study, including due to the sizeable declines in cohort fertility that have been observed in many European countries (Frejka and Calot 2001; Frejka 2008). In this study, it is hard to say why there are no material differences between adult immigrants from South and East Europe and child migrants from the same origins, which could relate to adaptation, selection, or childhood socialisation.

## Discussion

Although previous research has shown that immigrant fertility differentials vary by country of birth, this study develops new knowledge by demonstrating that there is considerable heterogeneity in differentials, not only for different migrant groups, but also over their life course. Comparing all thirteen of the origins groups that are analysed here, it is clear that there is considerable heterogeneity among foreign-born women in the UK.

The analysis provides a deeper understanding of immigrant fertility in the UK by examining all stages of childbearing. In doing so, it goes beyond what might be learnt from analyses using alternative approaches. For the first time in the UK, we show that period TFRs for immigrant women (in all periods studied) are higher than estimates of completed fertility (for all cohorts studied). Our results also demonstrate, using longitudinal data for completed fertility profiles, that this is likely to be due to elevated fertility after immigration, (at least for adult immigrants from a large number of immigrant origin groups). These findings are new, although the existence of elevated fertility after arrival has also been proposed by prior research comparing the ASFRs of immigrants and natives in the UK before and after immigration (Dubuc 2012, 2016), or analysing cross sections of the childbearing life course shortly before and after migration (Robards and Berrington 2016).

As in prior research, it is important to be cautious in interpreting our evidence of elevated fertility after arrival. In particular, it is important not to compare fertility before and after migration in order to draw conclusions about the (causal) association between migration and fertility (Hoem and Kreyenfeld 2006a, b; Hoem and Nedoluzhko 2016). Nevertheless, this finding can be interpreted descriptively, even if it remains uncertain whether elevated fertility after arrival is driven by the selection process (e.g. hindering the migration of women with children), and/or the postponement of births to coincide with migration. This analysis is not able to say whether these births are truly postponed (e.g. compared with non-migrants at origin), or whether they occur just before or just after migration. Nevertheless, these results show that for many immigrants there is an increased rate of childbearing in the later stages of their reproductive lives, relative to the timing of native births, and on average births are postponed later for those who arrive at older ages. This finding is not universal, with an obvious exception being immigrants from Bangladesh who arrive aged 26–35. However, the results for Bangladeshi women may relate to a prevalence for early partnership, and distinct patterns of family reunification (Berrington 1994; Coleman et al. 2002; Iliffe 1978; Walvin 1984), although these explanations certainly warrant further research.

If elevated fertility after arrival does exist in the UK, then this implies that the analysis of period TFRs for evaluating immigrant fertility may not be appropriate for answering many research questions, including those that seek to estimate the differential contribution of immigrants to population growth. This conclusion aligns with previous research (Sobotka and Lutz 2011; Toulemon 2004, 2006), and when research is motivated by an interest in population growth, it may be preferable to instead focus on completed fertility. However, the analysis of completed fertility does not allow researchers to examine tempo differentials. One way to do this is to use a parity-specific approach (Kulu and Hannemann 2016; Kulu et al. 2017). An alternative is to follow the approach that is demonstrated here, which enables a comparison of quantum and tempo across the entire reproductive life course. Using this approach, we show that estimates based on period TFRs or completed fertility alone appear to mask the complexity of variation in differentials that are evident over the life course for different immigrant groups.

One of the important implications of these results is that, although there are a number of immigrant groups with higher completed fertility than natives, these all have different profiles. This suggests that their fertility should be studied separately wherever possible in future research. By comparison, the origin country groups that have a lower completed fertility than natives (which includes most of the high income countries), all have quite similar patterns of differentials to each other. This suggests that it may be reasonable to group them together, and that research on these origins might best be directed towards the early childbearing ages.

Of course, there are limitations to these findings, and the extent to which they can be generalised remains uncertain. It is important to note that these results are for particular cohorts of women who have completed their fertility, and the childbearing of women from later birth cohorts may well be different. Future research could extend the approach taken here and investigate cumulative fertility differentials for cohorts of women who have not yet completed their childbearing. It is also important to consider that this issue of generalisability not only applies to immigrants but also to natives. These results are with reference to UK-born women, who may themselves have particular fertility patterns, such as higher levels of teenage childbearing, as compared with women in other immigrant destinations. Another limitation is that these results are based on those immigrants who remain in the UK, thereby excluding those who emigrate or die between arrival and time of survey. Added to this are some specifics of the sample, such as the exclusion of immigrants arriving aged 36 and over that may further affect the representativeness of these results.

Despite these limitations, and the need to be cautious when interpreting the findings, these results for the UK also provide indicative evidence for and against some prominent hypotheses that have been used to explain migrant fertility behaviour. For example, the existence of positive differentials across the life course for child migrants from Pakistan and Bangladesh, including with respect to their completed fertility, suggests that their fertility preferences may be culturally entrenched. As noted, this result might be due to socio-economic ‘entrenchment’, rather than the influence of ‘culture’. Nevertheless, in contrast to most other origin groups, this is tentative evidence against childhood socialisation (although arrival before age 16 does not guarantee that the majority of socialisation took place in the UK), and suggests that these groups may be worthwhile to study in future research. However, it is also important to note that, on its own, the absence of fertility differentials for child migrants is not enough to demonstrate childhood socialisation. A more reliable test of childhood socialisation would require data that more closely examines the interaction between migration background and childhood context.

Regardless of the explanation for these patterns, these results highlight the fact that it may be inappropriate to make generalisations about the quantum and tempo of immigrant fertility, as compared with natives. Conclusions about immigrant fertility differentials are very likely to depend upon the way that fertility is measured and the groups that are investigated. Similarly, these results show that the composition of the immigrant population will be very important in determining immigrant fertility differentials, and this includes the composition of the samples that are analysed. For example, the analysis of samples that include women who have not yet finished their childbearing may have a material impact on any conclusions about the magnitude of differentials, both now and in the future.

## Appendix

See Tables 2, 3, 4, and 5.

**Table 2:** Hypotheses and explanations for first generation fertility

Hypothesis/explanation	Adult migrants^a^	Child migrants^a^	Later generations
Disruption	Interruption of fertility before or after migration, followed by a recovery of childbearing to previous or higher levels	Limited effect as long as migration is before childbearing begins	No prediction
Anticipation^b^	Usually suggests a postponement of fertility until after migration, although some have suggested that women may seek to expedite births prior to migration	Limited effect as long as migration is before childbearing begins	No prediction
Elevated fertility^b^	Increase in fertility shortly after migration	Limited effect as long as migration is before childbearing begins	No prediction
Family formation/interrelation of events	The impact of migration depends on other events, in particular partnership, and is usually predicted to affect birth timing	Limited effect as long as migration is before childbearing begins	No prediction
Selection	Immigrants are different from the population at origin, and this may affect all aspects of their fertility	Selection is much weaker, but may have some impact on fertility	No prediction
Reverse causality	Those intending to migrate are less likely to do so if they have children, thereby leading to a selection of migrants who are more likely to give birth after migration	Limited effect as long as migration is before childbearing begins	No prediction
Legitimacy	Birth timing is driven by desire to obtain citizenship	No prediction	No prediction

^a^Adult migrants, as opposed to child migrants, are those whose age at migration is above a given threshold (e.g. 16-years-old)

^b^Anticipation and elevation are often described as part of the disruption hypothesis, but they are distinguished here in order to help clarify the distinctions between them

**Table 3:** Hypotheses with predictions for later generations

Hypothesis/explanation	Adult migrants^a^	Child migrants^a^	Later generations
Adaptation^b^	Fertility converges over the life course, presumably due to changes in birth timing	No differences in fertility compared with natives or the native norm, especially for the youngest arrivals	No differences compared with natives or the native norm
Intergenerational assimilation^c^	Fertility of origin is maintained	Some convergence of one or more aspects of fertility across generations, either the level or timing of births, or both	Convergence across generations until there are no differences compared with natives or the native norm
Childhood socialisation^c^	Fertility of origin is maintained (due to the country context of socialisation)	Generational convergence likely to be complete, dependent on child environment	Generational convergence likely to be complete, dependent on child environment
Cultural entrenchment	Fertility of origin is largely maintained	Differential fertility may be sustained due to exposure to origin subcultures	Differential fertility may be sustained due to exposure to origin subcultures
Minority group status	Unclear, but fertility depends upon the status of minority	Unclear, but fertility depends upon the status of minority	Unclear, but fertility depends upon the status of minority
Social characteristics	Fertility depends upon the characteristics of the migrant	Fertility depends upon the characteristics of the migrant	Fertility depends upon the characteristics of the descendant

^a^Adult migrants, as opposed to child migrants, are those whose age at migration is above a given threshold (e.g. 16-years-old)

^b^Adaptation is often referred to as a form of individual convergence or individual assimilation

^c^In some studies, it would seem that intergenerational assimilation and childhood socialisation are hard to distinguish, so this table follows the most prevalent use

**Table 4:** Description of the analytical sample (women born 1942–1971)

Category		UK-born (native)		Foreign-born	
		Frequency	%	Frequency	%
Country of birth					
UK	(All)	9724	100		
	(Ancestral natives)	8660	89		
	(2nd generation)	1064	11		
Ireland				83	6
India				174	13
Pakistan				132	10
Bangladesh				78	6
Jamaica				97	7
Other Caribbean				67	5
New Zealand, Australia, USA, Canada				61	4
Northern and Western Europe				81	6
Southern and Eastern Europe				49	4
North Africa and Middle East				73	5
Western and Central Africa				121	9
Eastern and Southern Africa				187	14
East Asia				89	6
Other countries				80	6
Age at migration					
Under 16 (child migrant)				449	33
16–25				551	40
26–35				372	27
Any children born before migrated?					
Yes				277	20
No				1095	80
Total sample		9724	100	1372	100

*Source*: UKHLS data (author’s analysis)

**Table 5:** Age-specific characteristics

Category	UK-born (native)		Foreign-born	
	Frequency (or mean)	%	Frequency (or mean)	%
Mean number of children (unweighted)				
At age 20	0.25		0.24	
At age 30	1.48		1.62	
At age 40	2.03		2.44	
At age 50 (or oldest age)	2.06		2.49	
At age 50 (histories only)^b^	2.04		2.47	
Mean number of children (weighted)^a^				
At age 20	0.25		0.18	
At age 30	1.47		1.40	
At age 40	2.02		2.15	
At age 50 (or oldest age)	2.05		2.19	
At age 50 (histories only)^b^	2.03		2.17	
Partnership status at age 40				
No partner	1896	19	258	19
Cohabiting	894	9	75	5
Married	6934	71	1039	76
Partnership history at age 40				
Never partnered	529	5	96	7
Cohabited, never married	837	9	84	6
Married, never cohabited	5273	54	909	66
Married, has cohabited	3085	32	283	21
Total sample	9724	100	1372	100

*Source*: UKHLS data (author’s analysis)

^a^Calculated taking account of the survey design

^b^Resident children estimated from history questions, rather than household questionnaire

## References

[CR1] Abbasi-Shavazi MJ, McDonald P (2000). Fertility and multiculturalism: Immigrant fertility in Australia, 1977–1991. International Migration Review.

[CR2] Adserà A, Ferrer A (2014). Factors influencing the fertility choices of child immigrants in Canada. Population Studies.

[CR3] Adserà A, Ferrer A (2014). The fertility of married immigrant women to Canada. International Migration Review.

[CR4] Adserà A, Ferrer AM, Sigle-Rushton W, Wilson B (2012). Fertility patterns of child migrants age at migration and ancestry in comparative perspective. The ANNALS of the American Academy of Political and Social Science.

[CR5] Agresti A (2002). Categorical data analysis.

[CR7] Alders, M. (2000). *Cohort fertility of migrant women in the Netherlands: Developments in fertility of women born in Turkey, Morocco, Suriname, and the Netherlands Antilles and Aruba*. Department of Population Division, Statistics Netherlands, Paper for the BSPS-NVD-URU Conference, Utrecht, 31 August–1 September, 2000.

[CR8] Allen, K., & Warrell, H. (2013, August 8). Baby boom drives UK population growth. *Financial Times*. Retrieved from http://www.ft.com/cms/s/0/7a42c2de-0008-11e3-9c40-00144feab7de.html#axzz3WIAOxn9O. Accessed 8 Aug 2013.

[CR9] Andersson G (2004). Childbearing after migration: Fertility patterns of foreign-born women in Sweden. International Migration Review.

[CR10] Andersson G, Scott K (2005). Labour-market status and first-time parenthood: The experience of immigrant women in Sweden, 1981–97. Population Studies.

[CR11] Andersson G, Sobolev B (2013). Small effects of selective migration and selective survival in retrospective studies of fertility. European Journal of Population/Revue Européenne de Démographie.

[CR12] Bagavos C, Tsimbos C, Verropoulou G (2007). Native and migrant fertility patterns in Greece: A Cohort approach. European Journal of Population/Revue Européenne de Démographie.

[CR13] Batson CD (2013). Contemporary fertility patterns and first-birth timing among Mexican-origin women. Hispanic Journal of Behavioral Sciences.

[CR14] BBC. (2013). Booming birth rate “a strain on NHS midwifery services.” *BBC News*. Retrieved from http://www.bbc.co.uk/news/health-21120593. Accessed 4 Apr 2015.

[CR15] Bean FD, Swicegood G (1985). Mexican American fertility patterns.

[CR16] Bélanger, A., & Gilbert, S. (2006). The fertility of immigrant women and their Canadian-born daughters. In *Report on the demographic situation in Canada, 2002* (pp. 127–151). Statistics Canada.

[CR17] Berrington A (1994). Marriage and family formation among the white and ethnic minority populations in Britain. Ethnic and Racial Studies.

[CR18] Berrington, A. (2018) Expectations for family transitions in young Adulthood among the UK second generation. Working paper, Southampton, GB: CPC.

[CR19] Bongaarts J, Feeney G (1998). On the quantum and tempo of fertility. Population and Development Review.

[CR21] Castro-Martín T, Cortina C (2015). Demographic issues of intra-European migration: Destinations, family and settlement. European Journal of Population.

[CR23] Coleman DA (1994). Trends in fertility and intermarriage among immigrant populations in Western Europe as measures of integration. Journal of Biosocial Science.

[CR24] Coleman DA (2009). The demographic effects of international migration in Europe. Oxford Review of Economic Policy.

[CR25] Coleman DA, Compton P, Salt J, Haug W, Compton P, Courbage Y (2002). Demography of migrant populations: The case of the United Kingdom. The demographic characteristics of immigrant populations.

[CR28] Dale A, Shaheen N, Kalra V, Fieldhouse E (2002). Routes into education and employment for young Pakistani and Bangladeshi women in the UK. Ethnic and Racial Studies.

[CR29] Daley PO (1998). Black Africans in Great Britain: Spatial concentration and segregation. Urban Studies.

[CR30] Dormon, O. (2014). *Childbearing of UK and non*-*UK born women living in the UK*—*2011 Census data*. Office for National Statistics. Retrieved from http://www.ons.gov.uk/ons/dcp171766_350433.pdf. Accessed 5 Feb 2014.

[CR31] Dubuc S (2012). Immigration to the UK from high-fertility countries: Intergenerational adaptation and fertility convergence. Population and Development Review.

[CR32] Dubuc S, Champion PT, Falkingham J (2016). Immigrants and ethnic fertility convergence in the UK: The role of global fertility transition and intergenerational social integration. Population change in the United Kingdom.

[CR34] Easton, M. (2012, July 16). What is the UK’s optimum population? *BBC*. Retrieved from http://www.bbc.co.uk/news/uk-18854762. Accessed 8 Feb 2013.

[CR38] Fokkema T, de Valk H, de Beer J, Van Duin C (2008). The Netherlands: Childbearing within the context of a “Poldermodel” society. Demographic Research.

[CR39] Foner N (2009). Gender and migration: West Indians in comparative perspective. International Migration.

[CR40] Forste R, Tienda M (1996). What’s behind racial and ethnic fertility differentials?. Population and Development Review.

[CR42] Frejka T (2008). Overview Chapter 2: Parity distribution and completed family size in Europe. Demographic Research.

[CR43] Frejka T, Calot G (2001). Cohort reproductive patterns in low-fertility countries. Population and Development Review.

[CR44] Frejka T, Sardon J-P (2007). Cohort birth order, parity progression ratio and parity distribution trends in developed countries. Demographic Research.

[CR45] Friedlander D, Goldscheider C (1978). Immigration, social change and cohort fertility in Israel. Population Studies.

[CR46] Garssen J, Nicolaas H (2008). Fertility of Turkish and Moroccan women in the Netherlands: Adjustment to native level within one generation. Demographic Research.

[CR49] Goldscheider C, Uhlenberg PR (1969). Minority group status and fertility. American Journal of Sociology.

[CR50] Goldstein S, Goldstein A (1981). The impact of migration on fertility: An `own children’ analysis for Thailand. Population Studies.

[CR51] Goldstein, S., & Goldstein, A. (1983). Migration and fertility in Peninsular Malaysia. *A rand note, prepared for the agency for international development*, *[N*-*1860*-*AID]*. Retrieved from http://www.rand.org/pubs/notes/N1860.html.

[CR52] Grant J, Hoorens S, Sivadasan S, van het Loo M, DaVanzo J, Hale L (2004). Low fertility and population ageing: Causes consequences and policy options.

[CR53] Hampshire K, Blell M, Simpson B (2012). Navigating New socio-demographic landscapes: Using anthropological demography to understand the ‘persistence’ of high and early fertility among British Pakistanis. European Journal of Population/Revue Européenne de Démographie.

[CR54] Harbison SF, Weishaar M (1981). Samoan migrant fertility: Adaptation and selection. Human Organization.

[CR55] Harper S, Hamblin K (2014). International handbook on ageing and public policy.

[CR56] Haug W, Compton P, Courbage Y (2002). The demographic characteristics of immigrant populations.

[CR57] Hervitz HM (1985). Selectivity, adaptation, or disruption? A comparison of alternative hypotheses on the effects of migration on fertility: The case of Brazil. International Migration Review.

[CR58] Hill LE, Johnson HP (2004). Fertility changes among immigrants: Generations, neighborhoods, and personal characteristics. Social Science Quarterly.

[CR59] Hoem JM, Kreyenfeld M (2006). Anticipatory analysis and its alternatives in life-course research (1). Demographic Research.

[CR60] Hoem JM, Kreyenfeld M (2006). Anticipatory analysis and its alternatives in life-course research (2). Demographic Research.

[CR61] Hoem, J. M., & Nedoluzhko, L. (2014). *Pre*-*and post*-*migration fertility* (Stockholm Research Reports in Demography). Stockholm University. Retrieved from http://www.suda.su.se/SRRD/SRRD_2014_18.pdf.

[CR62] Hoem J, Nedoluzhko L (2016). The dangers of using “negative durations” to estimate pre- and post-migration fertility. Population Studies.

[CR63] Hornsby-Smith MP, Dale A (1988). The assimilation of Irish immigrants in england. The British Journal of Sociology.

[CR64] Horsfield G, Chappell R (2005). International migration. Focus on people and migration.

[CR65] Iliffe L (1978). Estimated fertility rates of Asian and West Indian immigrant women in Britain, 1969–74. Journal of Biosocial Science.

[CR66] Kahn JR (1988). Immigrant selectivity and fertility adaptation in the United States. Social Forces.

[CR68] Kulu H (2005). Migration and fertility: Competing hypotheses re-examined. European Journal of Population/Revue Européenne de Démographie.

[CR69] Kulu H, González-Ferrer A (2014). Family dynamics among immigrants and their descendants in Europe: Current research and opportunities. European Journal of Population.

[CR70] Kulu H, Hannemann T (2016). Why does fertility remain high among certain UK-born ethnic minority women?. Demographic Research.

[CR71] Kulu H, Hannemann T, Pailhé A, Neels K, Krapf S, González-Ferrer A, Andersson G (2017). Fertility by birth order among the descendants of immigrants in selected European countries. Population and Development Review.

[CR72] Lübke C (2015). How migration affects the timing of childbearing: The transition to a first birth among Polish women in Britain. European Journal of Population.

[CR74] Mayer J, Riphahn RT (2000). Fertility assimilation of immigrants: Evidence from count data models. Journal of Population Economics.

[CR76] Milewski N (2007). First child of immigrant workers and their descendants in West Germany: Interrelation of events, disruption, or adaptation?. Demographic Research.

[CR77] Milewski N (2010). Fertility of immigrants: A two-generational approach in Germany.

[CR78] Milewski N (2011). Transition to a first birth among Turkish second-generation migrants in Western Europe. Advances in Life Course Research.

[CR79] Murphy M, Voets S, Schoorl JJ, de Bruijn B (1995). The impact of migration on population composition: The British case. The demographic consequences of international migration.

[CR81] Mussino E, Van Raalte AA (2012). Immigrant fertility: A comparative study between Italy and Russia. International Migration.

[CR82] Ng E, Nault F (1997). Fertility among recent immigrant women to Canada, 1991: An examination of the disruption hypothesis. International Migration.

[CR83] Ní Bhrolcháin M (2011). Tempo and the TFR. Demography.

[CR85] ONS. (2011). *Fertility assumptions: 2010*-*based national population projections*. Retrieved from http://www.ons.gov.uk/ons/dcp171776_233451.pdf. Accessed 12 May 2012.

[CR86] ONS. (2012a). *International migrants in England and Wales, 2011*. Retrieved from http://www.ons.gov.uk/ons/dcp171776_290335.pdf. Accessed 25 Jan 2015.

[CR87] ONS. (2012b). *Population by country of birth and nationality report*. Retrieved from http://www.ons.gov.uk/ons/dcp171776_277619.pdf. Accessed 4 Mar 2013.

[CR89] ONS. (2013). *Immigration patterns of non*-*UK born populations in England and Wales in 2011*. Retrieved from http://www.ons.gov.uk/ons/dcp171776_346219.pdf. Accessed 23 Apr 2015.

[CR90] ONS. (2014). *Annual mid*-*year population estimates, 2013*. Retrieved from http://www.ons.gov.uk/ons/dcp171778_367167.pdf. Accessed 24 Apr 2015.

[CR91] ONS. (2016). *Vital statistics: Population and health reference tables*. Retrieved from https://www.ons.gov.uk/peoplepopulationandcommunity/populationandmigration/populationestimates/datasets/vitalstatisticspopulationandhealthreferencetables. Accessed 3 May 2017.

[CR92] Parrado EA (2011). How high is Hispanic/Mexican fertility in the United States? Immigration and tempo considerations. Demography.

[CR93] Parrado EA, Flippen CA (2012). Hispanic fertility, immigration, and race in the twenty-first century. Race and Social Problems.

[CR94] Parrado EA, Morgan SP (2008). Intergenerational fertility among Hispanic women: New evidence of immigrant assimilation. Demography.

[CR95] Peach C (2006). South Asian migration and settlement in Great Britain, 1951–2001. Contemporary South Asia.

[CR97] Pressat R (1985). The dictionary of demography (C. Wilson, Ed.).

[CR98] Rendall MS, Ball DJ (2004). Immigration, emigration and the ageing of the overseas-born population in the United Kingdom. Population Trends.

[CR100] Rendall MS, Salt J, Chappell R (2005). The foreign-born population. Focus on people and migration.

[CR101] Ritchey PN (1975). The effect of minority group status on fertility: A re-examination of concepts. Population Studies.

[CR102] Robards, J. (2012, June). *Estimating the fertility of recent migrants to England and Wales (1991*–*2001)*—*Is there an elevated level of fertility after migration?* Presented at the European Population Conference, Stockholm University.

[CR103] Robards J, Berrington A (2016). The fertility of recent migrants to England and Wales. Demographic Research.

[CR104] Rosenwaike I (1973). Two generations of Italians in America: Their fertility experience. International Migration Review.

[CR105] Ryder NB, Hiorns RW (1980). Components of temporal variations in American fertility. Demographic patterns in developed societies.

[CR106] Sedghi, A. (2014). Is it true there is a “startling” rise in the birth-rate of British Muslims? *The Guardian*. Retrieved from http://www.theguardian.com/news/datablog/2014/jan/10/rise-british-muslim-birthrate-the-times-census. Accessed 14 Jan 2014.

[CR107] Sevak, P., & Schmidt, L. (2008). *Immigrant*-*native fertility and mortality differentials in the United States*. Retrieved from http://deepblue.lib.umich.edu/handle/2027.42/61807. Accessed 2 Aug 2013.

[CR108] Sigle-Rushton W (2008). England and Wales: Stable fertility and pronounced social status differences. Demographic Research.

[CR109] Smallwood, S. (2002). The effect of changes in timing of childbearing on measuring fertility in England and Wales. *Population Trends* 109. HMSO: Office for National Statistics. 12643048

[CR110] Sobotka T (2004). Is lowest-low fertility in Europe explained by the postponement of childbearing?. Population and Development Review.

[CR111] Sobotka T (2008). Overview Chapter 7: The rising importance of migrants for childbearing in Europe. Demographic Research.

[CR112] Sobotka, T., & Lutz, W. (2011). Misleading policy messages derived from the period TFR: Should we stop using it? *Comparative Population Studies*, *35*(3). Retrieved from http://www.comparativepopulationstudies.de/index.php/CPoS/article/view/54.

[CR113] StataCorp (2009). Stata Statistical Software: Release 11.

[CR114] Statistics New Zealand (2012). Demographic trends: 2011.

[CR115] Stephen EH, Bean FD (1992). Assimilation, disruption and the fertility of Mexican-origin women in the United States. International Migration Review.

[CR116] Toulemon, L. (2004). Fertility among immigrant women: new data, a new approach. *Population and Societies*, *400*. Retrieved from http://www.ined.fr/en/publications/pop_soc/bdd/publication/540/.

[CR117] Toulemon, L. (2006). *Fertility among immigrant women in France: New Data, a new approach*. Prepared for Population Association of American 2006 annual meeting, Los Angeles, California, March 30–April 1, 2006.

[CR118] Toulemon, L., & Mazuy, M. (2004). *Comment prendre en compte l’âge à l’arrivée et la durée de séjour en France dans la mesure de la fécondité des immigrants?* INED. Retrieved from http://www.ined.fr/fichier/t_publication/1053/publi_pdf1_120.pdf. Accessed 23 Oct 2013.

[CR119] Tromans N, Natamba E, Jefferies J (2007). Have women born outside the UK driven the rise in UK births since 2001?. Population Trends.

[CR120] University of Essex. (2011). Institute for Social and Economic Research and National Centre for Social Research, Understanding Society: Wave 1, 2009–2010 [computer file]. 2nd Edition. Colchester, Essex: UK Data Archive [distributor], November 2011. SN: 6614. 10.5255/UKDA-SN-6614-2.

[CR122] Waller L, Berrington A, Raymer J (2014). New insights into the fertility patterns of recent Polish migrants in the United Kingdom. Journal of Population Research.

[CR123] Walvin, J. (1984). *Passage to Britain: Immigration in British history and politics*. Penguin in association with Belitha Press.

[CR125] Wilson B, Kuha J (2017). Residential segregation and the fertility of immigrants and their descendants. Population, Space and Place.

[CR126] Young CM (1991). Changes in the demographic behaviour of migrants in Australia and the transition between generations. Population Studies.

[CR127] Zarate A, de Zarate AU (1975). On the reconciliation of research findings of migrant-nonmigrant fertility differentials in urban areas. International Migration Review.

[CR128] Zumpe, J., Dormon, O., & Jefferies, J. (2012). *Childbearing among UK born and non*-*UK born women living in the UK*. ONS. Retrieved from http://www.ons.gov.uk/ons/dcp171766_283876.pdf. Accessed 25 Oct 2012.

